# Identifying children with Cystic Fibrosis in population-scale routinely collected data in Wales: A Retrospective Review

**DOI:** 10.23889/ijpds.v5i1.1346

**Published:** 2020-08-11

**Authors:** R Griffiths, DK Schlüter, A Akbari, R Cosgriff, D Tucker, D Taylor-Robinson

**Affiliations:** 1 Swansea University Medical School, Swansea University; 2 Department of Public Health and Policy, University of Liverpool, Liverpool L69 7ZX; 3 Health Data Research UK; 4 Administrative Data Research Wales; 5 Cystic Fibrosis Trust, One Aldgate, London EC3N 1R; 6 Public Health Wales, Capital Quarter 2, Tyndall Street, Cardiff. CF10 4BZ\break † Joint First Authors

## Abstract

**Introduction:**

The challenges in identifying a cohort of people with a rare condition can be addressed by routinely collected, population-scale electronic health record (EHR) data, which provide large volumes of data at a national level. This paper describes the challenges of accurately identifying a cohort of children with Cystic Fibrosis (CF) using EHR and their validation against the UK CF Registry.

**Objectives:**

To establish a proof of principle and provide insight into the merits of linked data in CF research; to identify the benefits of access to multiple data sources, in particular the UK CF Registry data, and to demonstrate the opportunity it represents as a resource for future CF research.

**Methods:**

Three EHR data sources were used to identify children with CF born in Wales between 1^st^ January 1998 and 31^st^ August 2015 within the Secure Anonymised Information Linkage (SAIL) Databank. The UK CF Registry was later acquired by SAIL and linked to the EHR cohort to validate the cases and explore the reasons for misclassifications.

**Results:**

We identified 352 children with CF in the three EHR data sources. This was greater than expected based on historical incidence rates in Wales. Subsequent validation using the UK CF Registry found that 257 (73%) of these were true cases. Approximately 98.7% (156/158) of individuals identified as CF cases in all three EHR data sources were confirmed as true cases; but this was only the case for 19.8% (20/101) of all those identified in just a single data source.

**Conclusion:**

Identifying health conditions in EHR data can be challenging, so data quality assurance and validation is important or the merit of the research is undermined. This retrospective review identifies some of the challenges in identifying CF cases and demonstrates the benefits of linking cases across multiple data sources to improve quality.

## Introduction

There is increasing interest in using routinely collected, population-scale, electronic health record (EHR) administrative data, in order to conduct research. At population level these data sources can provide large volumes of data and a broad longitudinal view of a condition over a period of time [1-3]. In Wales, the Secure Anonymised Information Linkage (SAIL) Databank is the national repository of anonymised, person based, linkable data [4,5]. It holds routine EHR data from primary and secondary health care for about 5 million people from the turn of the century, including the active population of about 3.1 million people. Earlier data are available but at lower quality.

SAIL data are held within the privacy protecting UK Secure Research Platform (UKSeRP) based at Swansea University [6]. The SAIL Databank has a strict set of policies, structures and control practices in place to protect privacy through a matching, anonymisation and encryption process. The anonymisation process is conducted by the NHS Wales Informatics Service (NWIS) and involves splitting any identifiable data from the clinical data and assigning an Anonymised Linkage Field (ALF) to an individual based on a number of matching criteria. This enables the linkage of cases across multiple data sources that are acquired and linked into SAIL through matching via their ALF, and allows a broad longitudinal view of their conditions and treatments [7,8] .

Whilst there is considerable value to be gained from the use of routine EHR for research, it also poses a number of challenges [9-12]. These can occur because routine EHR rely on data collected for administrative purposes rather than clinical research, so identifying clinical outcomes or decisions can be more difficult. In addition, the coding systems used in the data may not be able to accurately convey the diagnostic conditions of interest. Accordingly, effective methodologies are required to both extract and validate these data to achieve high data quality and enable their use in clinical research [13].

In this study, we make use of a cohort of children with cystic fibrosis (CF) in Wales identified from electronic health records in SAIL from a previous study [14]. CF is a life-limiting, genetic disease caused by variants of the CF trans-membrane conductance regulator (CFTR) gene. It is characterised by the dysregulation of water and chloride ion transport across epithelial cell membranes leading to the build-up of mucus in various organs, in particular, those of the respiratory and digestive systems. CF is a classically inherited autosomal recessive condition whereby two faulty copies of the gene are necessary to develop CF. The severity of the disease can vary greatly and depends on a wide range of factors including the combination of mutations that someone inherits [15,16]. People with one faulty copy of the CFTR gene are carriers but do not display any symptoms. Approximately 1 in 2,500 babies born in the UK each year have CF [17] with 12 to 14 children with CF born in Wales every year [18].

CF is a rare condition and as with many rare diseases, access to enough cases for research can be problematic in the absence of access to national disease registries or where outcomes of interests are not captured in the registries [19]. We describe the challenges of accurately identifying children with CF in EHR and their validation against the UK CF Registry. We aim to establish a proof of principle and provide insight into the merits of linked data in CF research and the benefits of access to multiple data sources. In particular we highlight the benefit of linkage of routine data to the UK CF Registry and demonstrate the opportunity it represents as a resource for future CF research.

## Methods

This paper has been produced based on guidelines recommended for observational studies using administrative data [21-22].

### Data Sources

Several independent health data sources were available in the SAIL Databank in which it was possible to identify children with a CF diagnosis code. The Welsh Longitudinal General Practice (WLGP) data contains records of primary care General Practitioner (GP) events for people registered with a Welsh GP who contributes data to SAIL. Currently there is approximately 80% coverage of these data and the release made available to our study covered events from 1970 up to 2016. The Patient Episode Database for Wales (PEDW) data contains records of secondary care hospital admissions for all episodes of in-patient care, attending as a day-case or a longer term in-patient admission, in any NHS hospital. It also holds details for each episode of care, including diagnoses and treatment procedures. The release made available to our study covered admissions from 2000 to 2016.

The Congenital Anomaly Register Information Service (CARIS) data contains records of specialist registry data with details of babies and foetuses resident in Wales at time of birth, for whom an anomaly has been detected during pregnancy, birth or during the first year of life. Some of these anomalies, including CF, are picked up via a new-born screening test in the first week of birth, which was introduced in Wales in 1996 [23,24]. The release made available to our study covered children born from 1997 to 2013.

Furthermore, in order to identify children resident in Wales and extract and check background details, it was necessary to use other complementary data sources. These included the Welsh Demographic Service Dataset (WDSD), the National Community Child Health (NCCH) data, the Annual District Birth Extract (ADBE) data and the Annual District Death Extract (ADDE) data. For a description of these complementary data sources please see Supplementary Appendix 1.

The UK CF Registry records health data on consenting people with CF in England, Northern Ireland, Scotland and Wales and is managed by the Cystic Fibrosis Trust [25]. The data held by the Registry are collected at annual review visits at specialist CF care centres across the UK dating back to 1996. The data include a wide range of outcomes, complications and treatments, enabling research on a variety of issues in CF. It is estimated that the UK CF Registry captures 99% of the UK CF population [17]. The UK CF Registry was not available in the SAIL Databank at the time the initial CF cohort was derived [14]. However, at the end of 2018, the Cystic Fibrosis Trust gave permission for their data to be used in the SAIL Databank. The release made available to our study covered births to early 2018. 

### Method used to identify children in Wales with CF in the routine EHRs

Two different coding systems were required to identify people with CF in the EHR. The International Classification of Diseases Version 10 (ICD 10) is used to record diseases in PEDW and CARIS, whilst Read code Version 2 is used in WLGP. The list of ICD-10 and Read codes used to identify CF can be found in Supplementary Appendix 2. Structured Query Language (SQL) was adopted to extract all the data relating to the CF children.

In order to identify children resident in Wales, born between 1998 and 2015, an initial cohort of just under 600,000 children was extracted from the WDSD. This cohort was used as the basis for identifying those children in the WLGP, PEDW and CARIS data sources with a CF diagnosis code. This resulted in three independent groups of CF cases – one for each data source used to identify CF cases. The three groups were then cross matched and linked via their unique ALF and the data source of the diagnosis flagged. 

### Linkage with the UK CF Registry

At the end of 2018, the UK CF Registry was acquired into the SAIL Databank. Data acquisition by SAIL involves the negotiation and agreement of data owners and providers agreeing the use of their data in an anonymised format, for the use in research for the public good. Data that are acquired are then appropriately handled and managed through a split file process, that ensures identifiers and clinical information are handled and linked safely and securely [2,5,7].

Records from the three EHR data sources could then be linked to the cases in the UK CF Registry via their ALF. The availability of this ‘gold-standard’ in SAIL, now provided the opportunity to validate the original EHR cohort and identify the true cases. To this end, children born between January 1998 and August 2015 were identified within the UK CF registry and matched to our EHR cohort. In addition, it also provided an opportunity to identify Welsh resident children with CF that may have been unidentified previously. To determine Welsh residency in the UK CF Registry, we used LSOA (Lower layer Super Output Area) codes. LSOA is a geographic hierarchy level for statistical reporting and the highest granularity of identifiable geography permitted within the SAIL Databank in order to preserve privacy protection. In the 2001 Census listing there are 1,896 LSOAs in Wales, each with a population of about 1,500 people [26]. LSOA information is recorded for cases within the UK CF Registry. We report the positive predictive value of our method to identify CF cases in EHR. 95% confidence interval is based on the Gaussian approximation.

## Results

### Identification of children with CF in Wales using EHR

Using ICD-10 and Read codes in the three data sources the following numbers of cases were found: 252 in CARIS, 265 in PEDW and 244 in WLGP (see Figure 1).

**Figure 1: Illustration of the number of children with cystic fibrosis (CF) identified in electronic health records (EHR) in Wales, the number children in the UK CF Registry and the number of cases matched between the EHR cohort and the Registry. Sources used for identification of CF cases in EHR were Patient Episode Database for Wales (PEDW), Congenital Anomaly Register Information Service (CARIS) and Welsh Longitudinal General Practice (GP).**
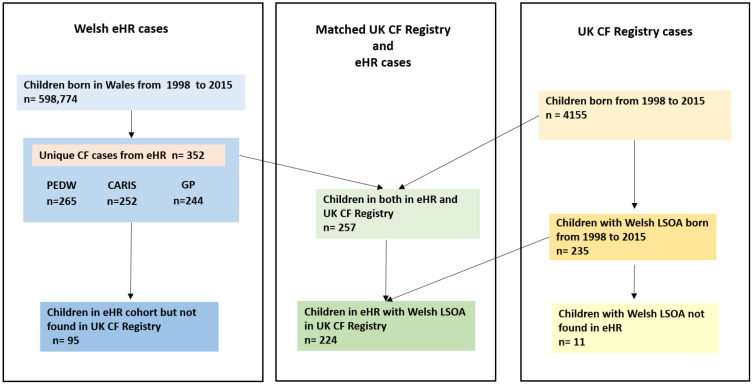


After matching the children on unique ALFs across all three data sources, we found a total of 352 unique children with CF diagnostic codes born between January 1998 and August 2015. This was higher than expected, given the incidence of CF in Wales, and it was assumed that some cases had been misclassified. As CF is a serious condition, often picked up at birth and likely to prompt frequent visits to a GP and possibly hospital admissions, there was an expectation that any child with CF would have records in multiple data sources, and the most likely true cases would be those with records in all three data sources.

### Validation of the EHR cohort against cases held within the UK CF Registry

Linkage of the 352 EHR cases with the UK CF Registry found 257 matched cases indicating that 95 cases identified in the EHR (27%) were potential false positives. Our method for identifying CF cases therefore had a positive predictive value of 0.73 (95% CI 0.68 – 0.78). Furthermore, 235 children were found with Welsh LSOA codes in the UK CF Registry, and with the exception of 11, all had been already identified in the EHR cohort.

Figure 1 illustrates the numbers of cases extracted from the respective data sources, and the numbers of cases that matched between the EHR and UK CF Registry data sources.

Approximately 99% (156/158) of EHR cases, with records in all three data sources, could be matched to individuals in the UK CF Registry. This supported our assumption that cases in this group would be the least likely to be misclassified. However, this group only captured 60% of the children we could match to the UK CF Registry. For individuals identified across two data sources, 87% (81/93) could be matched; these made up 32% (81/257) of all the children matched to the UK CF Registry. All individuals identified in CARIS and WLGP could be found in the Registry. Out of the individuals identified in CARIS and PEDW or PEDW and WLGP, 84% (42/50) and 89% (34/38) could be found in the Registry, respectively. Only 19.8% (20/101) of EHR cases, for whom we found a CF diagnosis code in just a single data source could be found in the UK CF Registry. The lowest proportion of true positives was in the group identified in CARIS (12.8% ( 5/39)) , followed by those identified in WLGP (13.9% ( 6/43) ); amongst children with a CF code only in PEDW 47% (9/47) could be matched (Figure 2 ).

**Figure 2: This diagram shows the number of children with CF codes in CARIS, PEDW and WLGP and the percentage of children matched to the UK CF Registry data. The table at the bottom of the figure gives the total number of cases identified in each EHR data source and the percentage of children found in the UK CF Registry.**
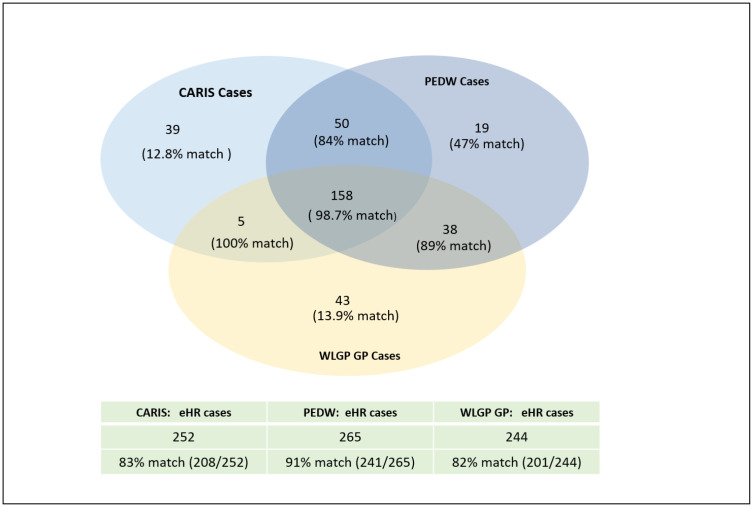


### Examination of the 95 false positive EHR cases

As the UK CF Registry captures 99% of the UK CF population [17], we suspect that most of the 95 cases identified in the EHR which were not found in the UK CF Registry were false positives. Within the group of children identified in two EHR sources but not found in the UK CF Registry, six children died during the study period, most within the first few years of life. In the group of cases found in both WLGP and PEDW, there was some evidence that what was being flagged was a ‘suspicion of’, or an’ investigation into CF’ rather than a diagnosis. In the group of cases found in CARIS and PEDW, some children (< 5) had a number of comorbidities. All the children identified in both CARIS and WLGP data sources were found in the UK CF Registry.

A little over 80% of all children found only in single data sources, were suspected to be wrongly identified with CF, and the predominant reason for misclassification appeared to be that CF was suspected but never confirmed in these children. The cases misclassified in CARIS had additional information within the records suggesting that a number of the children had been flagged as ‘carriers of CF’, having a ‘suspicion of CF’ or having ‘borderline sweat tests’. A small number (<5) of these children had also died and had complex congenital abnormalities. Cases misclassified in PEDW only, included children who also had complex health problems.

The biggest group of misclassified cases were found in the WLGP dataset. 14 were flagged as having been ‘seen in the CF clinic’ but had no further CF related records. Primary Practice is where most preliminary investigations for CF would take place and the most likely place for a new case, suspected or otherwise to be flagged up and recorded. Although there are Read codes for ‘suspected CF’, a simple CF diagnosis code seemed to be used in preference. 

### Examination of the eleven missing cases

The 11 children not identified in the EHR, were not found in any of the three datasets, with or without a CF diagnosis code. As they were not in CARIS, this implies they were not born in Wales and were not part of the Welsh new-born screening programme. The children may have moved into Wales at some date after the EHR cohort had been created.

## Discussion

We identified a cohort of children with Cystic Fibrosis (CF) in routinely collected data and validated these against a cohort of true cases. We showed that we could correctly identify 95% (224/235) of the true Welsh CF cases in electronic health records, however we had a high number of false positives (27% of the identified population). Our results highlighted that using multiple data sources is helpful in reducing the misclassification problem, and is particularly beneficial when a gold standard dataset is not available.

EHR data are of great benefit to research, but these data sources demand a good clinical understanding of the condition of interest and a good understanding of their administrative purposes. This includes how the data are collected, how they are used and coded for particular conditions and how frequently they are updated. There is an increasing acknowledgement that multiple record linkage , when used to combine information from a variety of digital health sources, is a powerful mechanism, and can generate rich comprehensive research data [27]. In this case, access to the routine health records in primary and secondary care, the data collected on congenital anomalies and the supplementary data collected in other supporting administrative data sources, were all available. Collectively, these were able to provide detailed information on disease conditions and treatments and the timings of events such as general interactions with services and provisions of care, potentially outside the main focus of CF treatment but affecting ongoing health.

There is an increasing interest in understanding health, social and economic consequences of CF beyond what is captured in the registries. This is highlighted by the increasing number of studies linking EHR to other CF Registries , for example to study the economic impact of CF [28] or to address detailed questions relating to in-hospital care of people with CF [29]. In this study, we have shown the importance of using multiple data sources to identify individuals with CF if a gold standard (e.g. a CF Registry) is not available. Using only one data source, for example hospital admissions data, will underestimate the number of cases and may bias the analyses. Although our method overestimated the number of CF children, the method managed to capture all but eleven of them, and showed good quality when using them in combination. The final validation of the cohort using a gold-standard demonstrated that the majority of cases identified in all three data sources, were also found in the UK CF Registry, thus confirming our assumption that multiple data sources can help improve the accurate identification of cases. Based on these results, we would recommend that any study of CF using EHR data without a gold standard, considers a range of sensitivity analyses which use case definitions based on the identification of cases in different combinations of data sources.

Identifying missing cases, such as the eleven not identified in the original EHR, can be a challenge. This occurred in our study when extracting and matching records from multiple data sources which were updated at different times and covered different time periods. For the small number of missing cases, a further investigation showed that CF records could be found for some, but only in much later versions of the health data and none appeared to be living in Wales at the time the EHR cohort was extracted. The SAIL Databank receives regular updated extracts of routine EHR data, and there is an agreement to receive further UK CF Registry updates in the future. Using the established coding classifications and the reproducible methods we have developed to identify and validate the CF cases, we can now maintain and update this derived CF EHR cohort for future research purposes.

Finally, for a condition like CF, there is an additional challenge when there is a need to record ‘suspected’ and ‘borderline’ cases, using the ICD-10 coding system, as it does not have a specific code to record either of these conditions. There are ICD-10 codes for identifying screening for genetic carriers and genetic susceptibility for disease, but these were not used for the EHR CF cohort and so ‘E84’ was used as a proxy. The Read codes seemed to suggest the opposite problem; there are a large number of codes for recording a CF condition, suspected or otherwise but given the wide choice, a simple CF diagnosis code was often chosen. The use of coding systems in a consistent manner and the limitations apparent in some coding systems, is a recognised concern in the use of EHR [10,30] and the reasons for the misclassified cases seemed consistent with this concern.

## Conclusion

Linked population level EHR offered a valuable resource for this research and the SAIL Databank provided a secure environment in which it was possible to link cases across multiple data sources. Although linking data in this manner can help reduce the risk of misclassification, access to a gold-standard will always be advantageous, as it will permit the verification of the true cases.

For future CF research, this also demonstrates the ability to link the UK CF Registry data to a derived EHR cohort, providing a more enriched set of data for analysis of the condition, which can now be maintained and updated using these established coding classifications and reproducible methods. These can also be used to identify and validate future CF cases, offering a complementary resource to the annual review data already collected by the UK CF Registry.

## Acknowledgments

We thank the UK CF Registry team and the UK CF centres and clinics for submitting data to the Registry. Our special thanks go to the people with cystic fibrosis and their families who have agreed for their UK CF Registry data to be used for research.

We would also like to thank the Cystic Fibrosis Trust for permission to acquire the UK CF Registry data into the SAIL Databank, providing proof-of-principle for UK wide linkage. In the future, this will allow researchers to apply for access to these linked anonymised UK CF Registry data within the SAIL Databank.

Many thanks also to the CARIS Team in Public Health Wales (PHW) for approval to use their data. It has been particularly valuable to have access to the new-born screening data and background congenital anomalies in CARIS. This has provided additional data about the CF cases which would not have been possible from PEDW or WLGP data alone.

DKS and DTR were supported by the Strategic Research Centre “EpiNet: Harnessing data to improve lives” funded by the Cystic Fibrosis Trust. DTR is funded by the MRC on a Clinician Scientist Fellowship (MR/P008577/1).

This work was supported by Health Data Research UK, which receives its funding from HDR UK Ltd (NIWA1) funded by the UK Medical Research Council, Engineering and Physical Sciences Research Council, Economic and Social Research Council, Department of Health and Social Care (England), Chief Scientist Office of the Scottish Government Health and Social Care Directorates, Health and Social Care Research and Development Division (Welsh Government), Public Health Agency (Northern Ireland), British Heart Foundation (BHF) and the Wellcome Trust.

This study makes use of anonymised data held in the SAIL Databank, which is part of the national e-health records research infrastructure for Wales. We would like to acknowledge all the data providers who make anonymised data available for research.

## Conflicts of interest

The authors declare they have no conflicts of interest.

## Ethics statement

The data used in this study are available in the SAIL Databank at Swansea University, Swansea, UK. All proposals to use SAIL data are subject to review by an independent Information Governance Review Panel (IGRP). Before any data can be accessed, approval must be given by the IGRP. The IGRP gives careful consideration to each project to ensure proper and appropriate use of SAIL data. When access has been approved, it is gained through a privacy-protecting safe haven and remote access system referred to as the SAIL Gateway. SAIL has established an application process to be followed by anyone who would like to access data via SAIL https://www.saildatabank.com/application-process. This study has been approved by the IGRP as project 0504.

## Supplementary Appendices

### Supplementary Appendix 1

#### Complementary Data Sources.

These datasets provided background details on the CF cases and assisted in the validation of the data extracted for the cohort:

The Welsh Demographic Service Dataset (WDSD) complements the WLGP events data and maintains longitudinal records of GP registrations and residence changes across Wales. It also records Welsh residents’ demographic details such as week of birth, gender and date of death where applicable. This was the means by which children resident in Wales were identified. This release covered births up to 2015.

The National Community Child Health data (NCCH): Each of the seven health boards in Wales collect records on maternal and child health, born, resident or treated in Wales after 1987. It includes a range of data items including birth details such as week of birth, gestational age, gender, multiple birth and breastfeeding indicators and enables a child to be linked to their mother (31). This release covered children born from 1997 to early 2016.

The Annual District Birth Extract (ADBE) and the Annual District Death Extract (ADDE). These records are provided by the Office of National Statistics (ONS), and contain details on week of birth and birth weight, and date of death, and cause of death respectively. This release covered the period up to 2015.

### Supplementary Appendix 2

**ICD10 and Read codes:** ICD10 codes used to identify CF in CARIS and PEDW data.

CF ICD 10 codes	Description
E84	Cystic fibrosis
E840	Cystic fibrosis with pulmonary manifestations
E841	Cystic fibrosis with intestinal manifestations
E488	Cystic fibrosis with other manifestations
E849	Cystic fibrosis, unspecified

**Table:** Read codes used to identify CF in GP data.

CF Read Code	Description
1264.	FH: Cystic fibrosis
66k..	Cystic fibrosis monitoring
66k0.	Cystic fibrosis annual review
9No7.	Seen in cystic fibrosis clinic
C370.	Cystic fibrosis
C3701	Meconium ileus in cystic fibrosis
C3702	Cystic fibrosis with pulmonary manifestations
C3703	Cystic fibrosis with intestinal manifestations
C3704	Arthropathy in cystic fibrosis
C3707	Liver dis due cystic fibrosis
C3709	Exacerbation cystic fibrosis
C370y	Cystic fibrosis other manifest
C370z	Cystic fibrosis NOS
